# Management of Asymptomatic Severe Hypertriglyceridemia With Oral Therapy Only: A Case Study Shedding Light on Treatment Approaches and Potential Complications

**DOI:** 10.7759/cureus.44567

**Published:** 2023-09-02

**Authors:** Md Bhuiyan, Orin Pramanik, Faraz Badar

**Affiliations:** 1 Internal Medicine, Sharon Hospital and Nuvance Health, Sharon, USA; 2 Internal Medicine, Vassar Brothers Medical Center, Poughkeepsie, USA; 3 Internal Medicine, University of Toledo Medical Center, Toledo, USA

**Keywords:** hypertriglyceridemia-induced pancreatitis, eruptive xanthoma, asymptomatic hypertriglyceridemia, insulin drip, severe hypertriglyceridemia

## Abstract

We present a rare case of a 52-year-old male with asymptomatic severe hypertriglyceridemia exceeding 11,000 mg/dL, managed initially with oral therapy without the need for an insulin drip or plasmapheresis. However, due to non-compliance at home, the patient subsequently developed pancreatitis requiring treatment with an insulin drip. He was discharged on a regimen of fenofibrate, rosuvastatin, and omega-3, with no further episodes of symptoms. Asymptomatic patients with severe hypertriglyceridemia and a low risk of developing symptoms can be safely managed through close monitoring, statin, fibrate therapy, and lifestyle modifications, but the risk of acute pancreatitis persists with elevated triglyceride levels of over 500 mg/dL and a marked increase in risk with a triglyceride level of greater than 880 mg/dL.

## Introduction

Severe hypertriglyceridemia (HTG) can be defined as a triglyceride level ≥1000 mg/dL (≥11.3 mmol/L) [1,2]. Severe HTG can be caused by primary genetic disorders or secondary factors such as alcohol use, poor diet, smoking, decreased physical activity, and certain medications like oral estrogen, tamoxifen, isotretinoin, propofol, and protease inhibitors [1]. In patients with triglyceride levels ≥1000 mg/dL has increased very low-density lipoprotein (VLDL) and chylomicrons [3]. The function of VLDL and chylomicron particles is to deliver triglycerides to the peripheral tissues. VLDL is believed to be atherogenic, but elevated chylomicrons pose a higher risk of acute pancreatitis [3]. Therefore, HTG increases the incidence of initial and recurrent acute pancreatitis [4]. Targeted therapy involving lipid-lowering agents and specific triglyceride-lowering medications aims to mitigate the risk of acute pancreatitis and reduce the likelihood of cardiovascular events. While symptomatic HTG is typically managed with interventions like an insulin drip or plasmapheresis in a hospital setting, there are no established guidelines for managing asymptomatic severe HTG. Here, we present an unusual case of a 52-year-old male with asymptomatic severe hypertriglyceridemia exceeding 11,000 mg/dL, managed using oral medication.

## Case presentation

**Figure 1:**
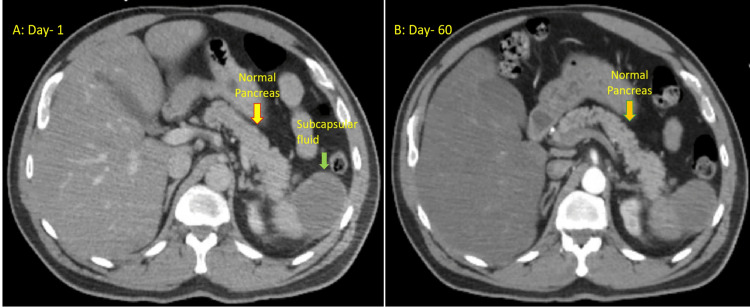
Image (A) showing normal pancreas on initial presentation. Image (B) showing repeat imaging 60 days after the initial presentation. Green arrow showing subcapsular fluid from previous trauma notable around the spleen.

A 52-year-old male with a past medical history of diabetes, a 50-pack year smoking history, obesity class-1, hyperlipidemia, and a recent traumatic chest injury from a motor vehicle with a long plate fixture to his clavicle and ribs, presented to the emergency department with chest discomfort and shortness of breath that began during a stress test. The patient has a significant family history of heart attack in his father at the age of 50. At the time of admission, the patient was hypoxic, requiring two liters per minute of supplemental oxygen via a nasal cannula. He had high-sensitivity troponin T levels of 17 and 14, and an EKG showed non-specific ST and T wave changes. A CT angiogram (CTA) of the chest ruled out pulmonary embolism (PE) but revealed some atelectasis. The lipid panel was significant for a triglyceride level of 11,078 mg/dL, cholesterol level of 976 mg/dL, and high-density lipoprotein (HDL) level of 6 mg/dL. The low-density lipoprotein (LDL) was unable to be calculated because of the hypertriglyceridemia. The patient's lipase level was 28 units/L, and a CT scan of the abdomen with intravenous contrast showed moderate subcapsular fluid replacing the majority of the expected splenic parenchyma from prior trauma but no pancreatitis (Figure 1A). The cardiologist evaluated the patient and determined that the chest pain was non-cardiac and likely caused by the chest trauma aggravated by sudden exercise. With rest, the patient's hypoxia and chest discomfort resolved. The patient reported previous triglyceride levels in the 500s but had not been compliant with medication. During the evaluation, the patient was completely asymptomatic and did not have nausea, abdominal pain, shortness of breath, chest pain, or blurred vision. There was a debate regarding the initiation of an insulin drip, but after consulting with two endocrinologists, it was decided not to initiate an insulin drip. His diet was changed to nothing by mouth (NPO) except medications, and he was started on Crestor and fenofibrate. The following day, his triglyceride level dropped from 11,078 mg/dL to 8006 mg/dL, cholesterol level decreased to 898 mg/dL, HDL level increased to 13 mg/dL, and LDL was <4 mg/dL. He was discharged home with prescriptions for Crestor, fenofibrate, icosapent ethyl, and omega-3 supplements. Follow-up appointments were scheduled with an endocrinologist and his primary care physician.

Unfortunately, the patient became ill with COVID-19 and failed to follow up with his doctors. Two months later, after not taking his medication, the patient presented to our emergency department with symptoms of nausea, vomiting, abdominal pain, and an eruptive rash on both his upper and lower extremities, as well as his posterior shoulders. The rash consisted of scattered small round papules with a yellowish center and faint pink erythema around the edges. A CT scan of the abdomen and pelvis showed an unremarkable pancreas (Figure 1B), while an abdominal ultrasound revealed diffuse echogenicity in the liver, consistent with fatty infiltration. There were no signs of cholelithiasis, ductal dilation, or gross abnormalities in the pancreas. The patient's aspartate transaminase (AST) level was 7 units/L, alanine transaminase (ALT) level was 18 units/L, alkaline phosphatase was 196 units/L, and total bilirubin was 6.7 mg/dL. Triglyceride levels were greater than 12,000 mg/dL, cholesterol level was 1109 mg/dL, HDL level was 5 mg/dL, LDL level was 48 mg/dL, and lipase level was 60 units/L. The patient was advised to be admitted to the hospital for the management of hypertriglyceridemia, abdominal pain, and closer monitoring. However, the patient strongly insisted on being discharged as his symptoms had resolved. The patient was educated about the risks associated with hypertriglyceridemia and the potential development of pancreatitis. Refills of his medication were provided, and the patient was recommended to immediately follow up with his primary care physician and endocrinologist.

**Figure 2:**
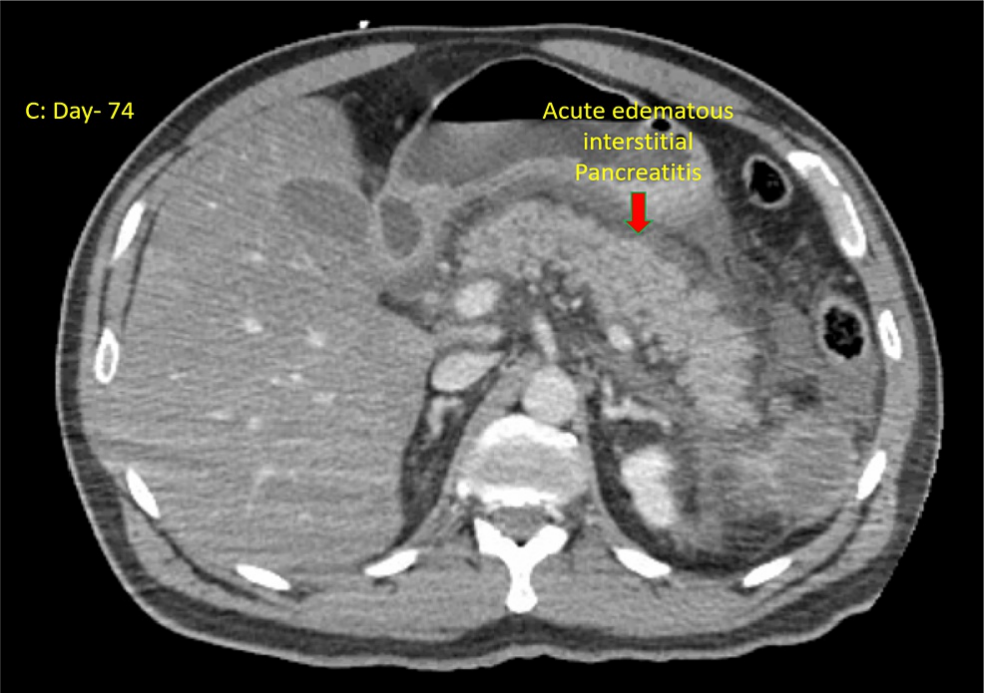
Acute edematous interstitial pancreatitis seen on a CT scan of the abdomen.

Two weeks later, the patient again presented to the emergency room with recurrent episodes of vomiting. The laboratory results showed a triglyceride level of 4425 mg/dL, cholesterol level of 645 mg/dL, HDL level of 6 mg/dL, LDL level of <4 mg/dL, and lipase level of 2061 units/L. A CT scan of the abdomen revealed acute edematous interstitial pancreatitis (Figure 2). The patient was promptly started on an insulin drip and aggressive hydration and was subsequently transferred to a tertiary care hospital for further management. Over the course of two days, the patient's symptoms began to improve. Five days later, the insulin drip was gradually tapered off after his triglyceride level dropped below 900, and the patient was transitioned to oral medications. He was discharged home on fenofibrate 145 mg, rosuvastatin 10 mg, and omega-3 1000 mg. Six months into follow-up, he had no further episodes of pancreatitis.

## Discussion

Untreated severe hypertriglyceridemia can lead to various complications, including acute pancreatitis, hyperviscosity syndrome, eruptive cutaneous xanthomas, lipemia retinalis, and long-term cardiovascular issues [5]. Primary HTG can be classified according to the Fredrickson classification system into familial chylomicronemia, familial combined hyperlipidemia, familial dysbetalipoproteinemia, familial hypertriglyceridemia, and primary mixed hyperlipidemia. The most common primary causes of HTG are familial combined hyperlipidemia and familial hypertriglyceridemia. Secondary causes of HTG involve lifestyle factors such as alcohol consumption, a diet high in saturated fats and refined sugars, excessive calorie intake, decreased physical activity, smoking, and medical conditions like obesity, uncontrolled diabetes, hypothyroidism, nephrotic syndrome, Cushing's syndrome, HIV-associated lipodystrophy, and pregnancy. Additionally, certain medications, including thiazide diuretics, non-selective beta blockers, atypical antipsychotics, and glucocorticoids, can contribute to mild to moderate HTG. Severe elevations in triglyceride levels can be attributed to the use of oral estrogen, tamoxifen, raloxifene, clomiphene, isotretinoin, cyclosporine, sirolimus, capecitabine, propofol, protease inhibitors, and interferon [2,3].

Patients with a prior history of pancreatitis are at an increased risk of developing pancreatitis with severe HTG. These high-risk patients may benefit from being monitored in a hospital and receiving treatment with statins, omega-3 fatty acids, fibrates, a very low-fat diet, avoiding refined carbohydrates, and alcohol consumption [3,6]. In cases where oral medications fail to improve triglyceride levels, patients may need an insulin drip or, in rare instances, plasmapheresis. Plasmapheresis is not readily available in all hospitals and requires trained specialists and technicians and access to a central venous line. Side effects of plasma exchange include dizziness, muscle spasms, arrhythmia, and hypotension. Administering an insulin drip necessitates significant resource utilization, including ICU or step-down unit care, hourly blood glucose checks, and a basic metabolic panel every four to six hours to monitor hypokalemia. There are a few case reports where asymptomatic HTG levels exceeding 10,000 mg/dL were treated with insulin drip and/or plasmapheresis [5,7-10]. In a retrospective chart review conducted by Berberich et al., 22 hospital admissions for acute pancreatitis secondary to severe HTG were analyzed. Ten cases were managed conservatively with NPO and intravenous hydration, while twelve cases received additional treatment with an insulin drip alongside NPO and intravenous hydration. Both groups exhibited a similar rate of decline in triglyceride levels [11]. Thus, asymptomatic low-risk patients with severe hypertriglyceridemia can be managed through close monitoring, statin, fibrate therapy, and lifestyle modifications [12].

Our patient had multiple secondary causes contributing to his HTG. Initially, he showed a remarkable response to dietary modifications and oral medications, obviating the need for an insulin drip. However, due to unforeseen circumstances and poor adherence to our recommendation, he subsequently developed acute pancreatitis and eruptive xanthomas as a result of poorly controlled HTG. It is important to highlight that these complications arose two months after the initial treatment. Had our patient followed up with his physician after the initial discharge and adhered to the recommended medication and lifestyle modifications, he would likely have experienced better outcomes.

## Conclusions

Asymptomatic patients with severe hypertriglyceridemia and a low risk of developing symptoms can be safely managed with statins, fibrates, and lifestyle modifications, as long as the patient can be closely monitored. However, it is important to note that there is still a risk of developing acute pancreatitis at any point during the treatment, particularly if the triglyceride level remains elevated above 880 mg/dL. Further research is needed to determine the long-term adequacy of oral therapy for managing asymptomatic severe hypertriglyceridemia.
